# Investigation of microstructural failure in the human cornea through fracture tests

**DOI:** 10.1038/s41598-023-40286-3

**Published:** 2023-08-24

**Authors:** Sai Naga Sri Harsha Chittajallu, Himanshu Gururani, Kwong Ming Tse, Subha Narayan Rath, Sayan Basu, Viswanath Chinthapenta

**Affiliations:** 1 Department of Mechanical and Aerospace Engineering, Indian Institute of Technology Hyderabad (IIT Hyderabad), Hyderabad, India; 2 Department of Mechanical and Product Design Engineering, Swinburne University of Technology, Melbourne, Australia; 3 Centre for Technology Innovation, LV Prasad Eye Institute, Hyderabad, India; 4 Department of Biomedical Engineering, Indian Institute of Technology Hyderabad, Hyderabad, India; 5 Prof. Brien Holden Eye Research Centre, LV Prasad Eye Institute, Hyderabad, India

**Keywords:** Biomedical engineering, Mechanical engineering, Tissues

## Abstract

Fracture toughness of the human cornea is one of the critical parameters in suture-involved corneal surgeries and the development of bioengineered mimetics of the human cornea. The present article systematically studied the fracture characteristics of the human cornea to evaluate its resistance to tear in the opening (Mode-I) and trouser tear mode (Mode-III). Tear experiments reveal the dependency of the fracture behavior on the notch size and its location created in the corneal specimens. The findings indicate lamellar tear and collagen fiber pull-out as a failure mechanism in trouser tear and opening mode tests, respectively. Experimental results have shown a localized variation of tear behavior in trouser tear mode and indicated an increasing resistance to tear from the corneal center to the periphery. This article demonstrated the complications of evaluating fracture toughness in opening mode and showed that the limbus was weaker than the cornea and sclera against tearing. The overall outcomes of the present study help in designing experiments to understand the toughness of the diseased tissues, understanding the effect of the suturing location and donor placement, and creating numerical models to study parameters affecting corneal replacement surgery.

## Introduction

The scarcity of donor corneas for the increased demands of corneal transplantation needs has propelled a quest for tissue-engineered corneal surrogates that could mimic a healthy cornea. In a physiologically healthy cornea, the mechanical properties play a significant role in maintaining the corneal geometry, transparency, and keratocytes functionality^1^. Corneal geometry influences the performance of the human eye by affecting its visual acuity. Understanding the cornea's geometrical stability in ocular conditions such as Ectasia in keratoconus^2,3^ and post-LASIK^4,5^ eye, and microstructural strengthening post-CXL^6–8^ requires knowledge of mechanical stiffness of the cornea. Therefore, the stiffness of the human cornea has been the most widely explored corneal mechanical property^8–13^.

On the other hand, the geometrical stability of the cornea in the presence of multiple sutures, such as in the case of the Penetrating Keratoplasty (PK) procedure^14^, depends on the strength of the cornea against the sutures. PK is commonly known as corneal transplantation, wherein the suture bite is equivalent to a geometrical defect that lowers the strength of the cornea at the bite location. The fracture toughness is the key parameter that determines the strength of any material against geometrical defects such as holes and cracks^15^. Therefore, the fracture toughness of the human cornea is of particular interest in the design of tissue-engineered cornea to benchmark its strength against sutures. Several reports show the use of tissue-engineered corneas or re-cellularized cadaveric corneas. However, they lack the corneal strength for suturing, making it difficult to find applications in actual patients^16–18^.

While stiffness is a well-documented mechanical property of the human cornea, its fracture properties have not been fully elucidated. However, multiple studies have been reported on the fracture behavior of other biological tissues, such as fetal membranes^19,20^, skin^21–23^, adipose^24^, etc., as per the authors' knowledge, only a single study ^25^ reported the time-dependent fracture toughness of the porcine cornea in trouser tear mode (Mode-III). Further, the tissue-engineered corneas have been shown to have adequate tear and suture retention strength for their utilization in animal models. However, to benchmark these corneal surrogates, the literature does not contain any data related to native human corneas.

In physiological conditions, the mechanical anisotropy of corneal tissue in the presence of suture bites displays a complex scenario of mixed loading (Mode-I and Mode III). The strains in the cornea at the suture bite are complex due to the combined effect of pulling forces generated by the suture and bursting pressure generated by IOP. The pulling force generated by the suture represents an opening mode, where the two surfaces of the cornea are being pulled apart (mode I) by the tensile force of sutures. However, simultaneously, the tensile load by the sutures in the presence of IOP creates a shearing effect to cause an out-of-plane cornea tear similar to a trouser tear (mode III). Hence it is essential to study the fracture toughness of the human cornea in both trouser tear and opening mode.

The primary objective of the present study is to characterize the tear phenomena in the corneal tissue and discuss its implications on the strength of the cornea against suturing. The native cornea exhibits mechanical anisotropy and is an inhomogeneous tissue. The present study intends to assess the influence of the inhomogeneous nature of the cornea on its fracture behavior. This will help clinicians and scientists to develop mechanically viable tissue surrogates. The present first-of-its-kind study investigates the fracture toughness of the human cornea in trouser tear and opening mode. In this investigation, the fracture behavior of the human cornea is analyzed for varying strain rates and notch length. While the varying strain rates help to study the viscous effects, the notch length sensitivity helps understand the local variation of fracture toughness arising from the anisotropic nature of the cornea, wherein the notch lengths are typically considered based on the geometry of PK. The knowledge of the localized variation clinically helps in selecting the best suture point according to the fracture toughness. Choosing the best suture position is closer to the clinical significance of corneal transplantation.

## Methods

Corneal specimens were initially tested under trouser tear and opening mode. One of the remnants of both fracture tests was used for microstructural analysis using scanning electron microscopy (SEM) imaging to study the tear surface morphology. The uniaxial tension tests were conducted on the second remnant of trouser tear tests to examine the relationship between fracture toughness and stiffness of the cornea. Finally, a statistical significance study was performed using a one-way ANOVA and f-statistics tests to understand the significance of various parameters.

### Materials

The specimens used in the present study were disease-free human cadaver corneoscleral buttons procured from the Ramayamma International Eye Bank, Hyderabad, India. Helsinki Protocols are followed while handling, storing, and disposing of the corneal tissues used for the testing, and details of ethical approvals are provided in the ethics declaration sub-section. The procured specimens were stored in McCarey-Kaufman (MK) medium at 4 degrees Celsius^26^. The inclusion criterion for the present investigation was central corneal thickness (CCT), typically considered a corneal rigidity descriptor^27^. The CCT of the corneas tested in the present investigation falls in the range of 517 ± 0.014 microns. A similar inclusion criterion for the mechanical testing of the corneal tissues can be found in the literature^9,28^.

All the specimens were thawed to room temperature prior to mechanical testing. The clinically relevant details of the corneas, such as age, pachymetry (corneal thickness), and endothelial cell density (ECD were also obtained from the eye bank. The age of the human corneas under study was 70 ± 12 years. The detailed specimen data is provided in the supplementary document**.**

### Specimen preparation

**Figure 1:**
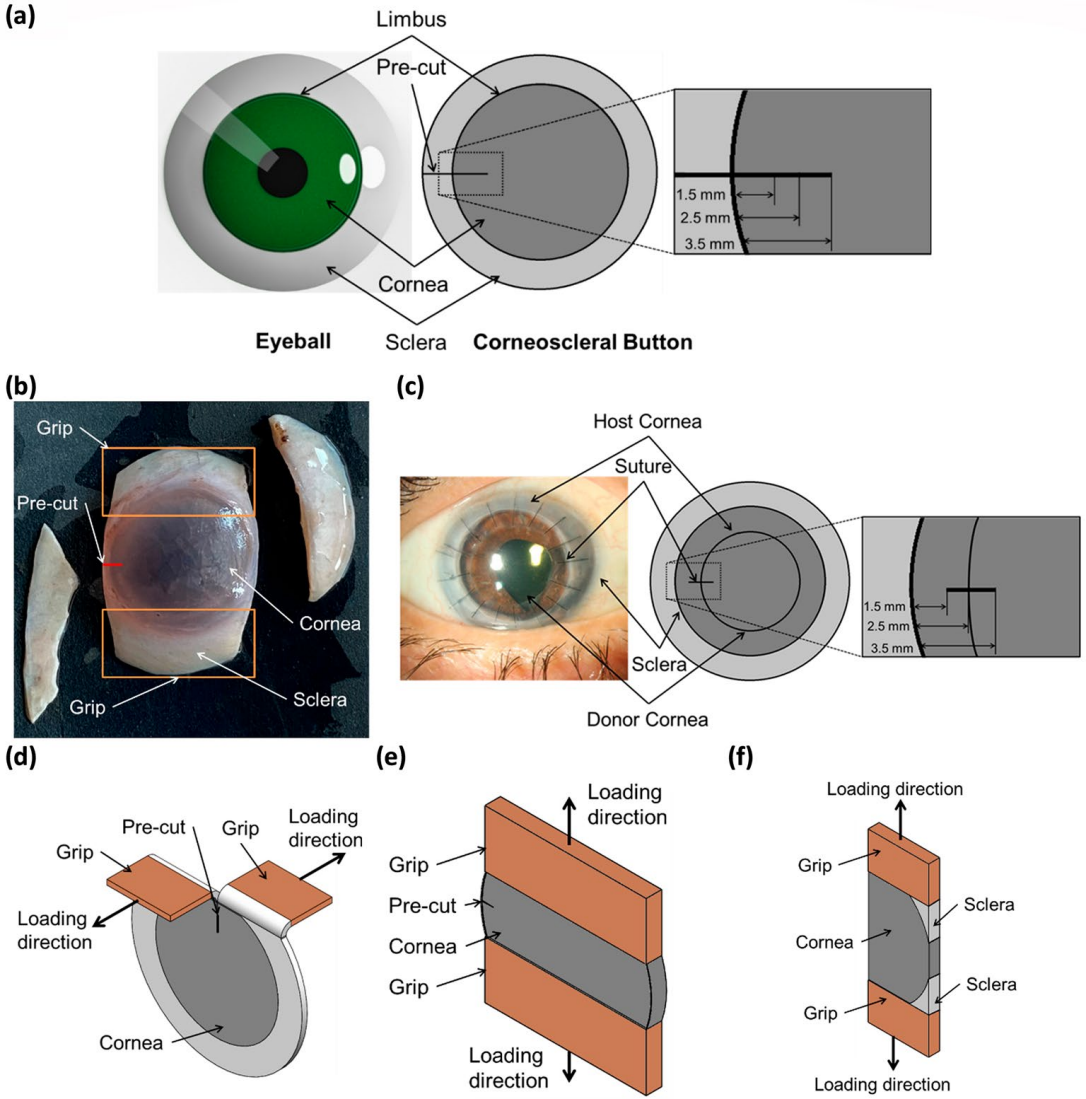
(**a**) A graphical representation of eyeball and corneoscleral button with the notch, (**b**) Opening mode specimen with remnants of the sclera, (**c**) Post PK cornea with the geometrical representation of sutures to define notch, (**d**) Trouser tear specimen loading configuration, (**e**) Opening mode specimen loading configuration and (**f**) loading configuration trouser tear remnants for stiffness evaluation.

Figure 1a shows a typical illustration of the corneoscleral button used in the present study. Unlike strip extensometry, where the corneal structure is completely disturbed due to specimen cutting, the whole corneoscleral buttons were used for mechanical testing in the present study. A single-edge notched specimen was prepared for testing the cornea in trouser tear mode (see Fig. 1b) and opening mode. For both tear tests, an edge notch was made using the surgical blade, as per the dimensions shown in Fig. 1a. The scleral rim was retained to ensure better gripping of the corneal specimens to the mechanical testing machine.

For the opening mode test, a section of the sclera adjacent and opposite to the notch was removed to ensure proper loading on the cornea (see Fig. 1e). The notch length was based on the suture bite dimensions used in penetrating keratoplasty (PK). Typically, in PK, the average outer diameter of the host and the donor cornea is 12 mm and 8 mm, respectively, with a suture length of 2 mm^14^. Based on the geometrical features of PK, the fracture toughness at the points of suture-donor, suture-host, and in-between the host and donor suture points were evaluated. Accordingly, three notch lengths were defined, i.e., 1.5 mm, 2.5 mm, and 3.5 mm, as illustrated in Fig. 1c.

### Trouser tear and opening mode tests

The tear and opening mode tests were conducted for the extension rates of 3, 30, and 300 mm/min to evaluate the strain-rate dependence of the fracture toughness. The specific strain rates were adopted from Tonsomboon et al.^25^ study to compare human and porcine cornea. All the tests were performed at room temperature on the universal testing machine (UTM) (Model 5944, Instron) equipped with a 50 N load cell. A set of customized grips were used to clamp and mount the specimens on UTM. Sandpaper of 800 grit size was placed in between the gripping surface and the specimen to avoid specimen slippage during the mechanical testing. During the tests, the specimens were irrigated intermittently with phosphate-buffered saline (PBS) solution to minimize specimen dehydration.

For trouser tear tests, the specimens were appropriately mounted to ensure minimum tilt of the tearing arms of the specimen with respect to grips and each other (see Fig. 1d). For opening mode tests, the notch was made parallel to the UTM arms to ensure loading of 4 ± 1 mm central part of the cornea, as shown in see Fig. 1e. Table 1 shows the test matrix showcasing the parameters for which the fracture toughness of the cornea was evaluated for each fracture mode. The strain rate dependency of fracture toughness in tearing and the opening mode was evaluated for a 2.5 mm notch length. The notch length dependency of fracture toughness in tearing mode was evaluated at a constant strain rate of 3 mm/min. Based on the work of Rivlin and Thomas^29^, the critical strain energy release rate ($T$) is given by:

1 $$ T = \frac{{W_{0} A_{0} - 2\lambda F_{0} }}{t} $$

where ${W}_{0}$ is the strain energy density in the arms of the specimen at ${F}_{0}$, $\lambda$ is the extension ratio in the arms of the specimen at ${F}_{0}$, ${A}_{0}$ is the area of the cross-section of the arms, and $t$ is the thickness of the specimen. Since the arms of the loaded specimens are very small compared to the specimen (see Fig. 1d), it can be assumed that negligible stresses are across the specimen arms while tearing. This fair approximation leads to $\lambda \cong 1$ and ${W}_{0}\cong 0$. Finally, the fracture toughness or energy of tearing in tearing mode^22,25,29^ is given by,

2 $$ T = \frac{{2F_{o} }}{t} $$

where, ${F}_{o}$ is the tearing load (peak load in load versus displacement curve) at which the crack begins to grow, and $t$ is the central corneal thickness. This technique to evaluate the critical strain energy release rate for tissue fracture is based on Rivlin and Thomas^29^. The technique assumes negligible stresses across the specimen arms while tearing, leading to the expression. The peak force ${F}_{o}$ is defined as the beginning of the catastrophic increase in the cut-length at uniform overall deformation of the specimen^29^. During this increase in length, the load in the load-extension diagram fluctuates around ${F}_{0}$ to form the plateau as described by Rivlin and Thomas^29^. In the present study, the central corneal thickness ($t$) is assumed constant throughout the specimen geometry. Although the cornea is typically thinner at the center than its periphery due to a greater diameter of collagen fibril in the periphery for the same inter-fibrillar spacing ^30^.

**Table 1:** Fracture toughness test matrix.

Test mode	Notch length (mm)	Strain rate (mm per min)	Sample size (n)	Age (years)	Thickness (mm)
Trouser tear	1.5	3	10	76 ± 10	0.517 ± 0.018
	2.5	3	10	69 ± 9	0.512 ± 0.012
		30	10	76 ± 8	0.516 ± 0.012
		300	10	67 ± 11	0.508 ± 0.008
	3.5	3	10	77 ± 7	0.526 ± 0.011
Opening (with notch)	2.5	3	10	71 ± 16	0.522 ± 0.014
		30	10	67 ± 6	0.510 ± 0.014
		300	10	72 ± 10	0.513 ± 0.013
Opening (without notch)	0	3	5	55 ± 24	0.521 ± 0.010
		30	5	56 ± 14	0.521 ± 0.010
		300	5	51 ± 10	0.524 ± 0.010

The fracture toughness in the opening mode needs to be obtained by calculating the difference between the strain energy of the opening mode specimens with and without the notch^19,31,32^. Without the notch, the mechanical testing of opening mode specimens resembles a simple uniaxial tension test (see Fig. 3h) with a specimen width of $w-a$. Where $a$ is the notch length, and $w$ is the width of the cornea^25^. In this test, the corneas were tested till rupture by varying the extension rates of 3, 30, and 300 mm/min. The sample size of specimens without a notch is limited to 5 due to their inconsistent failure location and the details discussed in the results and discussion sections.

### Uniaxial tension tests

Uniaxial tension tests were carried out to understand the role of the underlying microstructure of the cornea against the tearing. For this purpose, a torn specimen is loaded uniaxially along the direction of the tear (see Fig. 1f), and the relationship between tangential stiffness calculated at 3% strain is correlated with toughness. This methodology is adopted from a study by Purslow^22^. The remnants of the specimens after the trouser tear test were used for further tests and imaging. The simple uniaxial tension tests were conducted on one of the remnants to establish the relationship between fracture toughness and tangential stiffness along the crack propagation direction, as shown in Fig. 1f. At the same time, the other remnant was fixed immediately after the tear tests for SEM imaging. No particular strategy was followed in assigning the specimens for tension tests and SEM analysis.

The uniaxial tension tests were displacement controlled and were carried out at a 10 mm/min strain rate on the same UTM (see section "Trouser tear and opening mode tests"). The scleral rim opposite the torn edge of the cornea was removed (excluding the gripping locations) to eliminate the sclera's elastic effects from the cornea's mechanical response. The specimen dimensions (length 11 ± 1 mm and width 6 ± 0.5 mm) were recorded prior to the mechanical tests. An exponential function ($\sigma =a.{b}^{\epsilon }$) was used to fit the stress–strain data where the parameters a and b were evaluated. Further, the tangent stiffness (referred to as tangential stiffness here) was calculated at 3% strain as it corresponds to the physiological loading condition^33^ in the OriginPro 9 software (OriginLab Corporation, Northampton).

### SEM specimen preparation

The second remnant of the trouser tear and opening mode tests were fixed in a freshly prepared 2.5% glutaraldehyde (in 0.1M Phosphate Buffer Solution (PBS)) solution for microstructural analysis for 24 hours. Later, for secondary fixation (before imaging), the specimens were post-fixed in 1% osmium tetra-oxide (Sigma Aldrich) for 2 hours. The specimens were washed with PB three times and then subjected to gradual dehydration (20%, 35%, 50%, 75%, 95%, 100%) followed by dehydration with hexamethyldisilazane (HMDS, M/s. Sigma Aldrich) twice for 10 minutes each. The HMDS was decanted, and the treated specimens were allowed to air dry. The specimens were gold-sputtered and observed for microstructural analysis using JSM-7610F ultra high-resolution Schottky Field Emission Scanning Electron Microscope.

### Statistical analysis

One-way ANOVA tests were used to investigate the statistically significant difference between the fracture toughness of the specimens with varying strain rates and notch lengths for both opening and trouser tear with $\alpha =0.05$. To investigate the effect of strain rate, the experiments were carried out at a constant notch length of 2.5 mm with strain rates of 3, 30, and 300 mm/min for both trouser tear and opening mode. In another series of tests, the experiments were carried out at a constant strain rate of 3 mm/min for the notch lengths of 1.5, 2.5, and 3.5 mm for trouser tear mode to investigate the effect of notch length.

The values of fracture toughness (T) and tangential stiffness (E) were evaluated for each strain rate and notch length combination in the trouser tear test and reported in the supplementary. The significance of the relationship was determined with the help of linear regression analysis with 95% confidence interval bands. This correlation study was performed to quantify the relationship between fracture toughness at each strain rate and corresponding tangential stiffness. All the statistical analysis was performed in OriginPro 9.

### Ethics declaration

Helsinki Protocols are followed while handling, storing, and disposing of the corneal tissues used for the testing. The consent is sought from the kith/kin of the donor for medical research purposes. The current studies are performed with the ethical approval from the institutional review board from L V Prasad Eye Institute, Hyderabad (Ethics. Ref. No. 05-19-261, Dt. 14 May 2019), Institutional Ethics committee from IIT Hyderabad, Hyderabad (IEC Protocol No. IITH/IEC/2019/05/15, Dt. 2 May 2019), and Swinburne University of Technology Human Research Ethics Committee, Melbourne (Ref no. 20202695-3861, Dt. 04/03/2020).

## Results

### Tearing mode fracture response of the cornea

**Figure 2:**
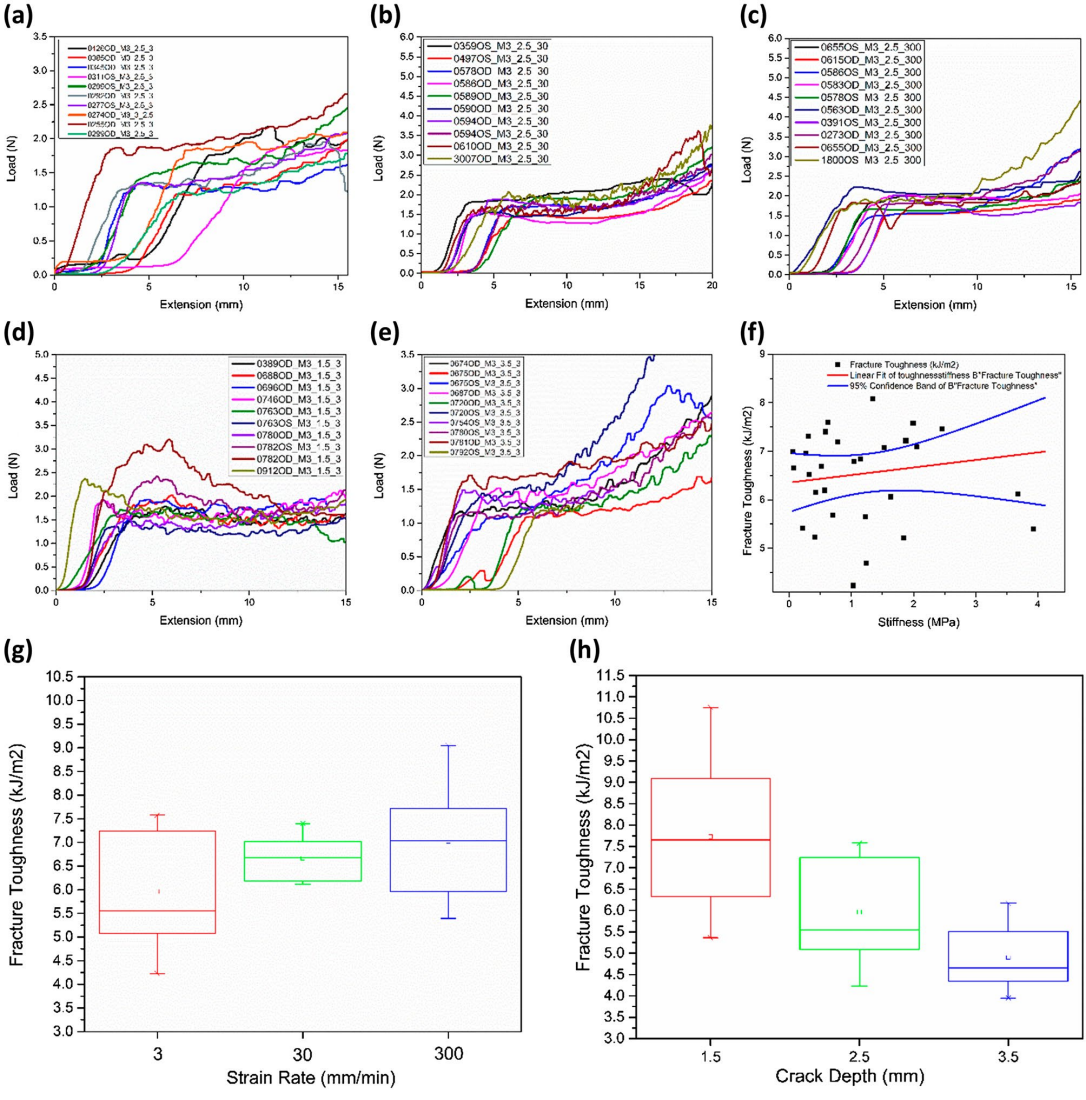
Load vs. extension plots for constant notch length of 2.5 mm and varying strain rates (**a**) 3 mm/min, (**b**) 30 mm/min, and (**c**) 300 mm/min, load vs. extension plots for constant strain rate 3 mm/min and for varying notch lengths (**d**) 1.5 mm, and (**e**) 3.5 mm, (**f**) fracture toughness vs. stiffness, experimental results of fracture toughness with respect to varying (**g**) strain rate, and (**h**) notch length.

The load versus extension graphs of the trouser tear tests are presented in Fig. 2a–e. Wherein Fig. 2a–c depicts material behavior for a 2.5 mm notch length at varying strain rates. Figure 2d, e shows the effect of varying notch length for the strain rate of 3 mm/min. The specimens have exhibited a similar tear phenomenon in trouser tear tests even though they are from different donors. The human cornea exhibited a non-linear tear propagation for all the testing parameters. The fracture toughness of the human cornea is found to be sensitive to the strain rate and notch length, as depicted in Fig. 2g, h. The average fracture toughness of the human cornea is found to vary from 5.87 ± 1.13 kJ/m^2^ at a 3 mm/min strain rate to 7.03 ± 1.19 kJ/m^2^ at a 300 mm/min strain rate for a 2.5 mm notch length. It is also found to vary from 7.73 ± 2.56 kJ/m^2^ at 1.5 mm notch length to 4.90 ± 0.48 kJ/m^2^ at 3.5 mm notch length for a 3 mm/min strain rate. The lowest fracture toughness is observed at 3 mm/min, and with the combination of 50 N load cell, the test is sensitive enough to capture the load variation during the tear propagation. Assuming a similar strain rate dependency from a 2.5 mm notch length and considering the sensitivity of the tests, the notch length dependency is restricted to a 3 mm/min strain rate. Additionally, soft tissues are reported to give reliable results at low strain rates^34,35^, and a low strain rate is also closer to representing the physiological conditions of ophthalmic tissues^36^.

The average fracture toughness is increasing with a rise in strain rates. However, the one-way ANOVA test shows the variation is statistically insignificant $(p = 0.068$, $p>\alpha$) Fig. 2g. On the other hand, the average fracture toughness diminishes with an increase in notch length, and the one-way ANOVA test shows that this variation is statistically significant ($p=0.0001$, $p<\alpha$) Fig. 2h. In tearing mode, the fracture toughness and the stiffness are not consistently correlated, as depicted in Fig. 2f, wherein the data points are randomly distributed beyond the 95% confidence band.

### Opening mode fracture response of the cornea

**Figure 3:**
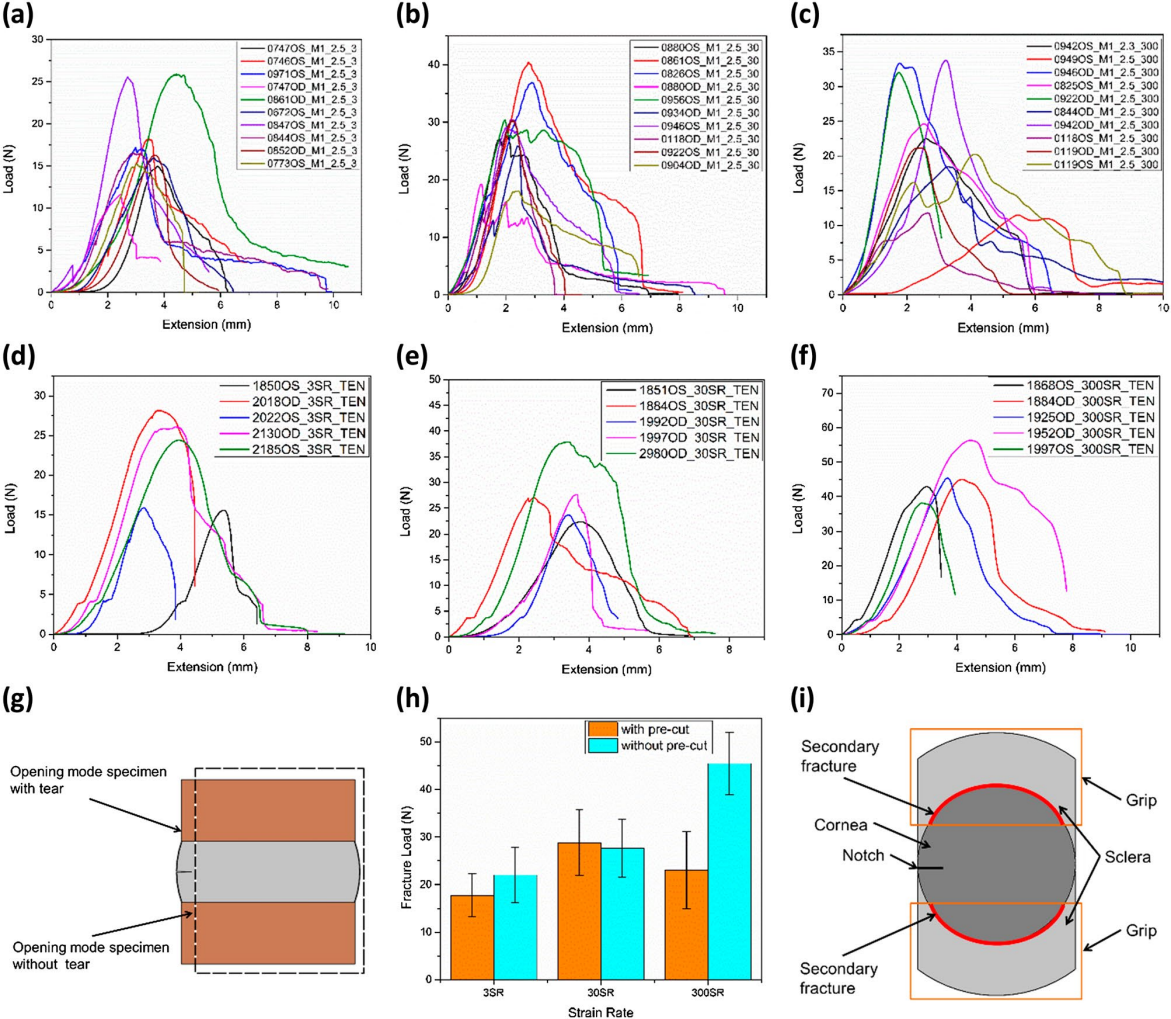
The load vs. extension plots of the human cornea in the opening mode for a constant notch length of 2.5 mm for varying strain rates at (**a**) 3 mm/min, (**b**) 30 mm/min, and (**c**) 300 mm/min, load vs. extension plots of the human cornea in the opening mode without a notch for varying strain rate at (**d**) 3 mm/min, (**e**) 30 mm/min, and (**f**) 300 mm/min, (**g**) graphical representation of specimen with and without a notch, (**h**) fracture load vs. strain rate comparing the opening mode tests with and without a notch, and (**i**) opening mode specimen with secondary fracture regions marked in red.

The mechanical behavior of the human cornea in opening mode with the notch is presented in Fig. 3a–c, and without the notch is presented in Fig. 3d–f. The difference between the specimen with and without the notch can be visualised in Fig. 3g. In opening mode tests, notch specimens have undergone crack blunting and necking before catastrophic fracture resulting in the crack propagation within the specimen, i.e., in the cornea. The average fracture loads vary from 17.73 ± 4.55 N at 3 mm/min, 28.81 ± 6.87 N at 30 mm/min, and 22.99 ± 8.14 N at 300 mm/min strain rate.

In specimens without notch due to their small size, the fracture/tear location was found to vary, i.e., mostly along the limbus and a few in the cornea. At the same time, their average fracture loads increase with an increase in strain rate from 22.03 ± 5.87 N at 3 mm/min to 45.47 ± 6.54 N at 300 mm/min strain rate. However, evaluation of corneal fracture toughness in opening mode mandates the fracture/tear formation in the cornea. In addition, no specific pattern is observed in the opening mode tear tests for varying strain rates with overlapping due to high variation, as visualized in Fig. 3h. Henceforth, the opening mode tear tests are confined to single notch length and without notch tests to only five specimens. Also, fracture loads are used to compare the mechanical load response of the specimens with and without the notch.

### Fractography of the human cornea

**Figure 4:**
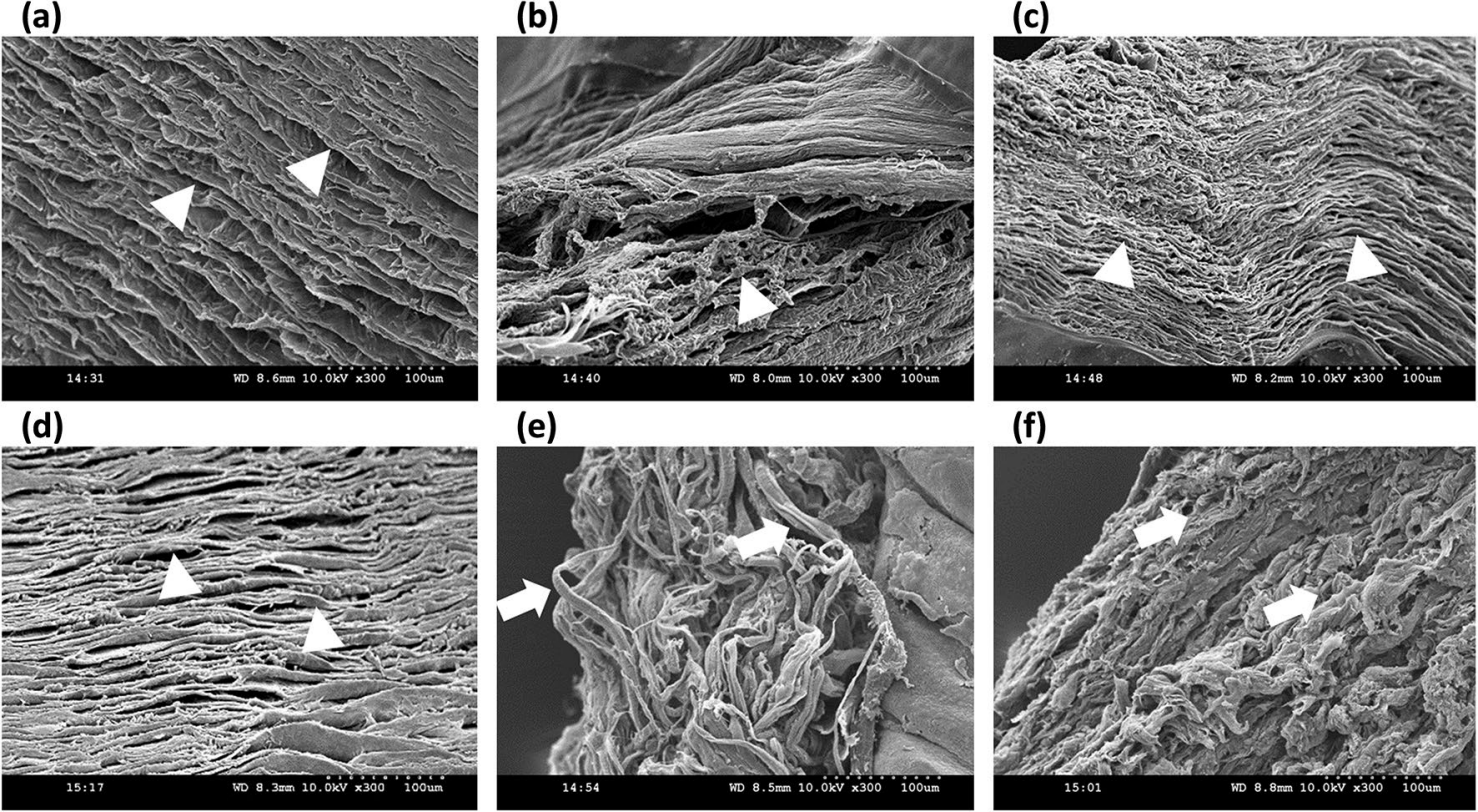
SEM images of the tear surface in tearing mode at the (**a**) notch, (**b**) in the mid-way, and (**c**) at the end of the tear, similarly the SEM image of the tear surface in opening mode at (**d**) notch, (**e**) in the mid-way, and (**f**) at the end of the tear, where triangle represents a lamellar tear, and arrow represents fiber pull out.

Figure 4a–f shows the scanning electron microscope images of the torn surfaces of the cornea. While the notch surface morphology is identical in both the modes (see Fig. 4a, d), they show spatial variation, i.e., the mid-way and end of the tear morphologies are quite different from one another. The fractured surface in the tearing mode specimens depicting lamellar tear, as shown in Fig. 4b, c. On the other hand, the fractured surfaces in opening mode specimens depicted collagen fiber bundle breakage, as presented in Fig. 4e, f.

## Discussion

**Figure 5:**
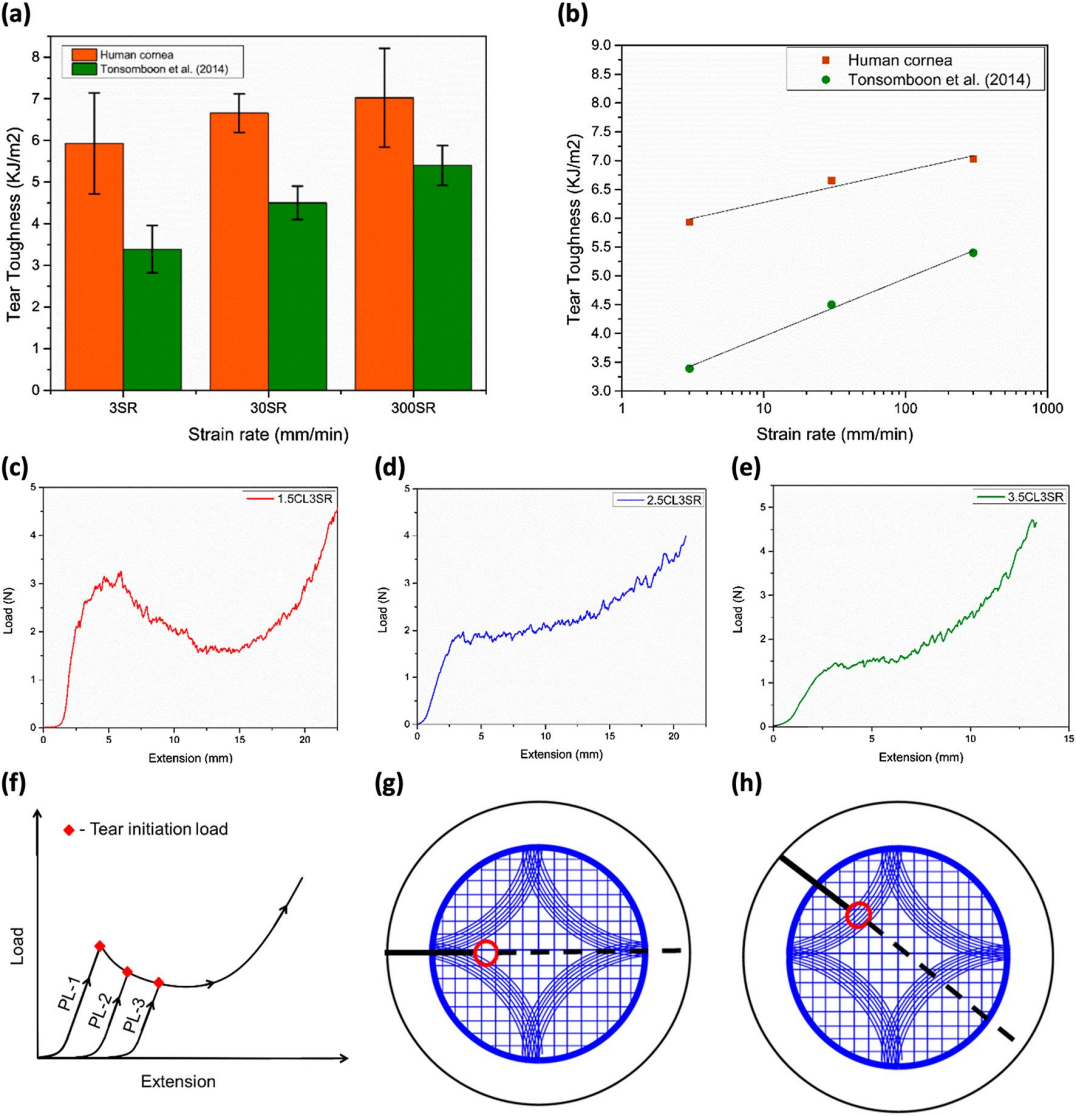
(**a**) Fracture toughness of the human cornea and porcine cornea by Tonsomboon et al.^25^, (**b**) average fracture toughness slop at various strain rates of human cornea and porcine by Tonsomboon et al., load vs. extension plots of the human cornea starting from notch length of (**c**) 1.5 mm, (**d**) 2.5 mm, and (**e**) 3.5 mm, (**f**) illustrates the overlapped load versus extension plots for all three notch lengths (NL-1 = 1.5 mm, NL-2 = 2.5 mm and NL-3 = 3.5 mm), representative images showing the stress concentration zone in here continuous line represents notch, red zone represents stress concentration zone, and the discontinuous line represents tear propagation path, collagen fiber distribution (Blue lines) according to Meek and the notch placed at (**g**) anchoring fibers, and (**h**) other than anchoring fibers.

The study investigated the fracture toughness of human corneas in trouser tear and opening mode and its dependence on strain rate and notch length. The results showed that the tear toughness of human corneas in trouser tear mode is higher than that of porcine corneas^25^ (see Fig. 5a). This is due to the difference in the thicknesses of the porcine and human cornea^37^. Additionally, the difference in the experimental protocol may influence the fracture toughness of the human and porcine corneas. Unlike the present study, where the MK medium was used to thaw and irrigate the tissue while testing, Tonsomboon et al.^25^ used a phosphate buffer solution to thaw porcine corneas, which may have affected the tissue hydration, its thickness^38^ and strain rate sensitivity^9,39^ (see Fig. 5b).

The tear toughness of the human cornea is found to be sensitive to the notch length (see Fig. 5c–e). The peak load corresponding to tear initiation varies with respect to the notch length. For example, the tear initiation load is maximum for a 1.5 mm notch length and minimum for a 3.5 mm notch length. It can be noticed that unlike the porcine cornea^25^, which exhibited a near-plateau curve after the initiation of the tear, the human cornea exhibited a moon-shaped load – displacement (L-D) behavior following the tear initiation. Interestingly, the L-D curve for 2.5 mm and 3.5 mm notch length appears to be a section of the L-D curve of 1.5 mm notch length. Based on the load variations captured during the tearing (see Fig. 5f), it is reasonable to infer that the fracture toughness of the human cornea is minimal in the central region (2.5 mm and 3.5 mm) and increases in the peripheral region (1.5 mm) of the cornea.

The behavior of the L-D curves for the human cornea can be correlated with the collagen fiber distribution established by Meek^40^. As per Meek’s model, the collagen fiber arrangement changes from orthogonal at the corneal center to circumferential towards the limbus (see Fig. 5g, h). This microstructural variability experienced by the tip of the propagating crack during tissue tear manifests in the form of a moon-shaped L-D curve for the human cornea. In contrast, the porcine corneas have predominantly superior-inferior aligned fibers resulting in different L–D curves^25,41^. The present study highlights the role of collagen fiber orientation in the fracture behavior of the human cornea.

**Figure 6:**
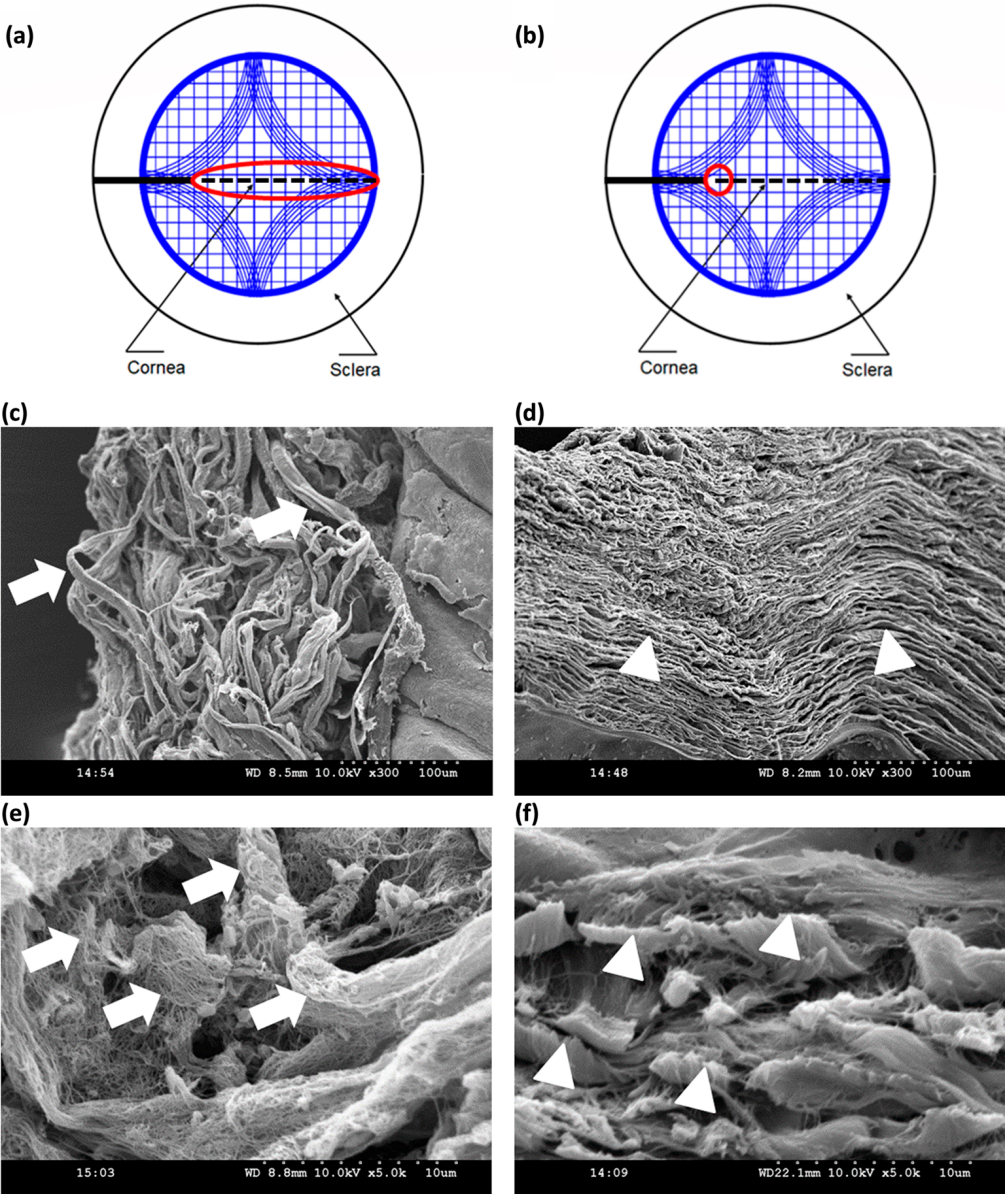
Representative images showing the stress concentration zone in (**a**) opening mode and (**b**) trouser tear mode, where the continuous line represents the notch, the red zone represents the stress concentration zone, and the discontinuous line represents the tear propagation path over the collagen fiber distribution according as per Meek ^40^, SEM images of tear surfaces at 300 magnification of (**c**) opening mode and (**d**) trouser tear mode, at 5k magnification of (**e**) opening mode and (**f**) trouser tear mode.

Interestingly, the peak load marking the crack propagation initiation in the mode I tear is comparatively higher than in mode III. The fiber recruitment during loading influences the tear mechanism of the tissue. In opening mode, all fibers across the sample width resist notch opening leading to higher values of load corresponding to crack initiation (see Fig. 6a). In contrast, only a small area near the notch tip resists tearing in mode III (Fig. 6b), consistent with that observed in rabbit and porcine skin^21,23^. The difference in the tear mechanism between the two modes can also be appreciated from the SEM images depicting the morphology of the fractured surfaces (see Fig. 6d, f). The lamellar tearing in mode III (see Fig. 6d, f) and fiber pull-out due and excessive crack blunting in mode I (see Fig. 6c, e) explain the observed difference in peak load for mode III and mode I, respectively.

**Figure 7:**
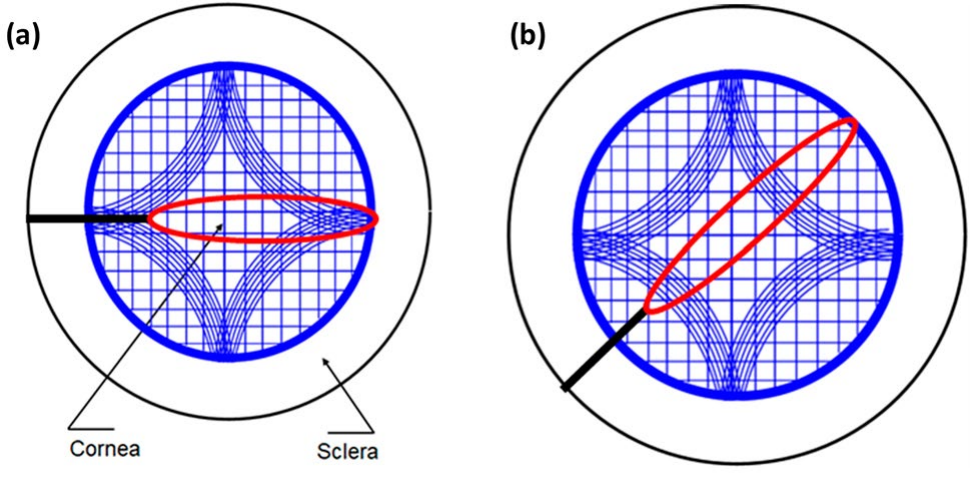
Collagen fiber distribution (Blue lines) according to Meek ^40^ and the notch placed at (**a**) anchoring fibers and (**b**) other than anchoring fibers, where the black line indicates notch and red area indicates stress concentration zone.

In the current study, the evaluated fracture toughness inherently possesses inter-subject dependency as the physiological orientation of the corneas is not known while collecting them from the eye bank. This is because the effective collagen fibers taking the load would vary with the location of the notch (see Fig. 7a, b). Hence, the notch length dependency in opening mode is restricted to the notch length of 2.5 mm. However, the opening mode tests provide insight into the overall tear resistance of the human cornea that is close to the physiological loading. At the same time, trouser tear tests provide the localized variation of the fracture toughness behavior.

The present study assesses the dependency of the tissue microstructure on its fracture behavior by conducting a correlation test between the fracture toughness and the stiffness of the cornea under uniaxial tension. For this purpose, the methodology of Purslow^22^ is applied here. The fracture toughness and tangent stiffness are not correlated (see Fig. 4f). This indicates that the non-homogeneity of the corneal microstructure results in different mechanical behavior in different meridians. Therefore, the correlation study failed to predict anything because of the random selection of notch locations for the fracture study.

The present investigation also provides some interesting findings. During mode I tear, a secondary crack was formed at the limbus of 12 out of 15 corneas in the opening mode tests without the notch, as illustrated in Fig. 3i. This might be due to the microstructure of the limbus that contains fibers running perpendicular to the loading direction in the junction between the cornea and the sclera^42^. These fibers offer lower resistance to the corneal tissue against the load in the opening mode. Moreover, an earlier study has reported that the thickness from the cornea to the sclera decreases and increases^43^. The cornea might be contracted laterally at a threshold load, leading to a stress concentration zone at the limbus and causing failure at the limbal junction.

**Figure 8:**
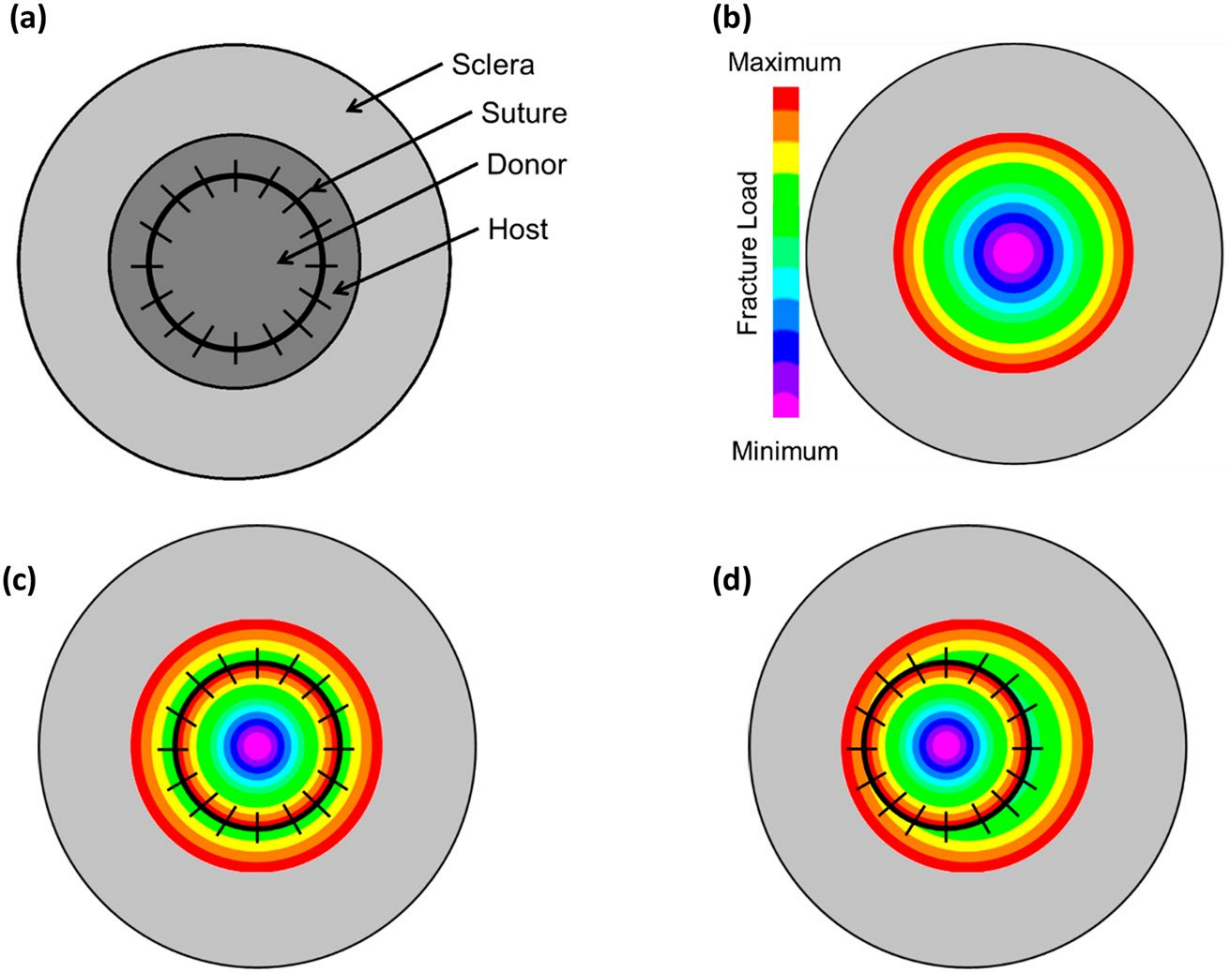
(**a**) Representation of post PK cornea with sutures, (**b**) fracture toughness in trouser tear mode mapped onto the human cornea, (**c**) ideal PK condition with sutures, and (**d**) when the location of PK is offset in one direction.

The results of the present study provide important insights into the biomechanical aspects of PK. For the donor tissue survival in PK, two parameters need to be studied, i.e., donor and host tissues resistance at suture bites and donor-host junction behavior. The present study gives a fair understanding of the donor and host tissue's resistance at suture bites with the help of fracture toughness. These results help in biomechanics simulations to understand various scenarios, such as donor placement changes in PK, how the diseased host will interact with the healthy donor, etc. The magnitude of the tension in the suture is one of the key parameters contributing to the corneas' post-PK topography. Figure 8a is a graphical representation of the PK cornea, and Fig. 8b shows the spatial representation of the fracture loads of the human cornea from L-D curves of trouser tear tests (see Fig. 5). For a perfectly aligned donor button, all the suture bites could have the same reaction forces as depicted in Fig. 8c. On the contrary, for a misaligned button, the suture tensions could vary based on their location, as illustrated in Fig. 8d, resulting in post-PK complications in refractive errors^44^ and intraocular pressure measurement^45,46^ as reported in the literature.

**Figure 9:**
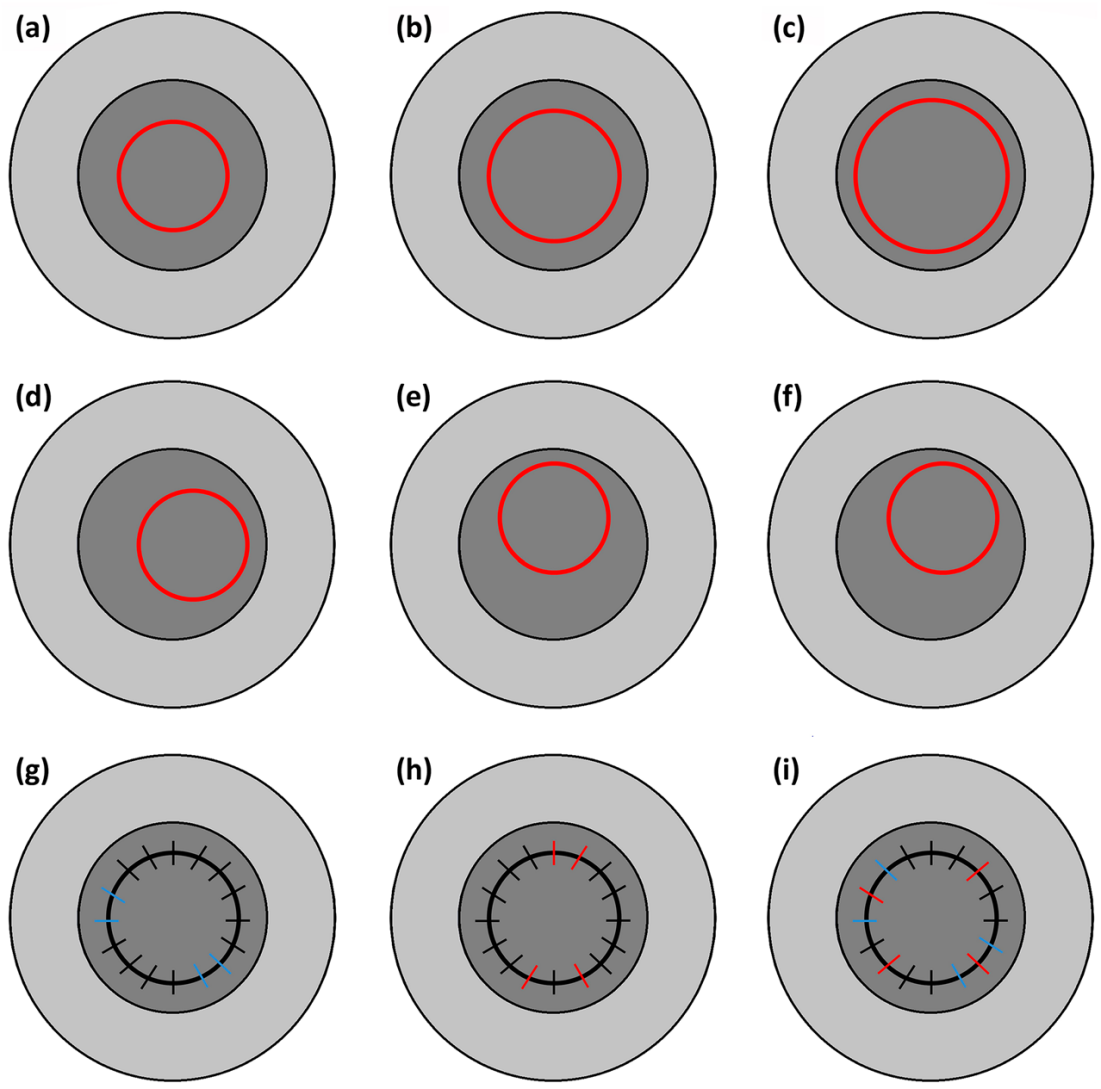
Size of PK as one of the parameters with an increase in diameter from (**a**) to (**c**), location of the PK as a varying parameter where the location is (**d**) towards one of the horizontal periphery, (**e**) towards one of the vertical periphery, and (**f**) towards one of the diagonal periphery, suture tension as on of the varying parameter where black lines across donor-host junction represent uniform tension sutures, blues represents loose sutures, and red represents tight sutures where the scenarios (**g**) PK with few loose sutures, (**h**) PK with few tight sutures, and (**i**) PK with combinations of loose, tight, and uniform sutures.

The results of the present study can be used to develop and validate rupture models for biomechanical simulations^47^. Numerical simulations of the host and donor with sutures by varying the mechanical properties of the host and donor, suture tension, host-donor orientation, and location of the donor will help to understand post-PK astigmatism^48^. The various scenarios of changes in the size of PK are depicted in Fig. 9a–c, the change of the PK location is illustrated in Fig. 9d–f, and varying suture tension in PK can be visualized in Fig. 9g–i.

The present study's findings lay a foundation for studying corneal tissue fracture behavior and developing biosynthetic materials for corneal surrogates. Relevant implantability parameters, such as suture retention strength^23^, pressure burst strength, and puncture resistance^49^, can aid in understanding physiological tear conditions. Tissue and suture adhesion also contribute to in-vivo sustainability, making the evaluation of physiologically relevant implantability parameters essential. The force required to tear the suture from the tissue is known as suture retention strength (SRS). While the suture retention strength of the native cornea has not been reported, multiple studies have characterized the SRS of bioengineered corneas and animal corneas^50^. Luo et al. reported a 5-6 N suture retention strength for the porcine scaffold and a tear resistance strength of 3.4 N for the porcine cornea^51^. Further experiments are underway to study the suture retention strength of the human cornea that closely replicates the in-vivo loading conditions.

The present first-of-its-kind study examines the fracture behavior of the human cornea and its dependency on the mode of fracture, strain rate, and notch length. The fracture toughness in trouser tear mode is independent of strain rate but the average fracture toughness is directly proportional to strain rate and inversely proportional to notch length. The opening mode reveals a ductile failure behavior and higher crack initiation loads than the tearing mode. Unlike the trouser tear, the opening mode tear revealed a catastrophic tear. The trouser tear mode displays a unique tear propagation with a low load at the centre, which increases towards the periphery. The Fractography reveals a lamellar tear in the trouser tear and fiber breakage in opening mode. These findings can help develop biosynthetic corneal surrogates, choose relevant suture points, and understand fracture toughness in pathological conditions.

### Supplementary Information

Supplementary Information.

## Data Availability

All data generated or analyzed during this study are included in this published article and its supplementary information files.
