# Supplementary material for: Investigation of microstructural failure in the human cornea through fracture tests
Source: Sci Rep. 2023 Aug 24;13:13876. doi: 10.1038/s41598-023-40286-3 (PMC10449857; doi:10.1038/s41598-023-40286-3)
Supplement: Supplementary file 1 — Supplementary Information. [file 41598_2023_40286_MOESM1_ESM.docx]

**Supplementary**

**Supplementary Table 1.** Trouser tear test specimen details

| **S. No.** | **Specimen** | **Strain Rate** | **Notch Length** | **Thickness** | **Age** |
| --- | --- | --- | --- | --- | --- |
|  |  | **(mm/min)** | **(mm)** | **(mm)** | **(Years)** |
| 1 | 0255OD | 3 | 2.5 | 0.521 | 62 |
| 2 | 0277OS | 3 | 2.5 | 0.500 | 60 |
| 3 | 0292OD | 3 | 2.5 | 0.500 | 78 |
| 4 | 0274OD | 3 | 2.5 | 0.500 | 45 |
| 5 | 0299OD | 3 | 2.5 | 0.518 | 81 |
| 6 | 0299OS | 3 | 2.5 | 0.514 | 81 |
| 7 | 0311OS | 3 | 2.5 | 0.500 | 70 |
| 8 | 0345OD | 3 | 2.5 | 0.519 | 75 |
| 9 | 0385OD | 3 | 2.5 | 0.552 | 65 |
| 10 | 0426OD | 3 | 2.5 | 0.500 | 74 |
| 11 | 0610OD | 30 | 2.5 | 0.500 | 85 |
| 12 | 0594OS | 30 | 2.5 | 0.503 | 70 |
| 13 | 0594OD | 30 | 2.5 | 0.521 | 70 |
| 14 | 0590OD | 30 | 2.5 | 0.514 | 70 |
| 15 | 0589OD | 30 | 2.5 | 0.535 | 75 |
| 16 | 0586OD | 30 | 2.5 | 0.526 | 85 |
| 17 | 0578OD | 30 | 2.5 | 0.526 | 70 |
| 18 | 0497OS | 30 | 2.5 | 0.500 | 95 |
| 19 | 0359OS | 30 | 2.5 | 0.532 | 69 |
| 20 | 3007OD | 30 | 2.5 | 0.500 | 71 |
| 21 | 0655OS | 300 | 2.5 | 0.500 | 55 |
| 22 | 0615OD | 300 | 2.5 | 0.514 | 90 |
| 23 | 0586OS | 300 | 2.5 | 0.526 | 85 |
| 24 | 0583OD | 300 | 2.5 | 0.502 | 80 |
| 25 | 0578OS | 300 | 2.5 | 0.521 | 70 |
| 26 | 0563OD | 300 | 2.5 | 0.514 | 67 |
| 27 | 0391OS | 300 | 2.5 | 0.500 | 60 |
| 28 | 0273OD | 300 | 2.5 | 0.506 | 55 |
| 29 | 0655OD | 300 | 2.5 | 0.500 | 55 |
| 30 | 1800OS | 300 | 2.5 | 0.500 | 60 |
| 31 | 0389OD | 3 | 1.5 | 0.500 | 60 |
| 32 | 0688OD | 3 | 1.5 | 0.500 | 90 |
| 33 | 0696OD | 3 | 1.5 | 0.500 | 73 |
| 34 | 0746OD | 3 | 1.5 | 0.500 | 84 |
| 35 | 0763OD | 3 | 1.5 | 0.530 | 85 |
| 36 | 0763OS | 3 | 1.5 | 0.544 | 85 |
| 37 | 0780OD | 3 | 1.5 | 0.516 | 89 |
| 38 | 0782OS | 3 | 1.5 | 0.500 | 65 |
| 39 | 0782OD | 3 | 1.5 | 0.552 | 65 |
| 40 | 0912OD | 3 | 1.5 | 0.532 | 65 |
| 41 | 0674OD | 3 | 3.5 | 0.535 | 80 |
| 42 | 0675OD | 3 | 3.5 | 0.500 | 70 |
| 43 | 0675OS | 3 | 3.5 | 0.548 | 70 |
| 44 | 0687OD | 3 | 3.5 | 0.518 | 76 |
| 45 | 0720OD | 3 | 3.5 | 0.518 | 80 |
| 46 | 0720OS | 3 | 3.5 | 0.529 | 80 |
| 47 | 0754OD | 3 | 3.5 | 0.532 | 75 |
| 48 | 0780OS | 3 | 3.5 | 0.532 | 89 |
| 49 | 0781OD | 3 | 3.5 | 0.536 | 65 |
| 50 | 0792OS | 3 | 3.5 | 0.514 | 92 |

**Supplementary Table 2.** Opening mode test specimen details

| **S. No.** | **Specimen** | **Strain Rate** | **Notch Length** | **Thickness** | **Age** |
| --- | --- | --- | --- | --- | --- |
|  |  | **(mm/min)** | **(mm)** | **(mm)** | **(Years)** |
| 1 | 0747OS | 3 | 2.5 | 0.500 | 92 |
| 2 | 0746OS | 3 | 2.5 | 0.518 | 84 |
| 3 | 0971OS | 3 | 2.5 | 0.500 | 75 |
| 4 | 0747OD | 3 | 2.5 | 0.524 | 92 |
| 5 | 0861OD | 3 | 2.5 | 0.527 | 60 |
| 6 | 0672OS | 3 | 2.5 | 0.521 | 76 |
| 7 | 0847OS | 3 | 2.5 | 0.500 | 60 |
| 8 | 0844OS | 3 | 2.5 | 0.527 | 88 |
| 9 | 0852OS | 3 | 2.5 | 0.575 | 25 |
| 10 | 0773OS | 3 | 2.5 | 0.525 | 60 |
| 11 | 0880OS | 30 | 2.5 | 0.500 | 63 |
| 12 | 0861OS | 30 | 2.5 | 0.528 | 60 |
| 13 | 0826OS | 30 | 2.5 | 0.526 | 80 |
| 14 | 0880OD | 30 | 2.5 | 0.500 | 63 |
| 15 | 0956OS | 30 | 2.5 | 0.547 | 65 |
| 16 | 0934OD | 30 | 2.5 | 0.500 | 65 |
| 17 | 0946OS | 30 | 2.5 | 0.500 | 68 |
| 18 | 0118OD | 30 | 2.5 | 0.500 | 81 |
| 19 | 0922OS | 30 | 2.5 | 0.500 | 62 |
| 20 | 0904OD | 30 | 2.5 | 0.500 | 63 |
| 21 | 0942OS | 300 | 2.5 | 0.526 | 78 |
| 22 | 0949OS | 300 | 2.5 | 0.526 | 75 |
| 23 | 0946OD | 300 | 2.5 | 0.500 | 68 |
| 24 | 0825OS | 300 | 2.5 | 0.531 | 82 |
| 25 | 0922OD | 300 | 2.5 | 0.500 | 62 |
| 26 | 0844OD | 300 | 2.5 | 0.521 | 88 |
| 27 | 0942OD | 300 | 2.5 | 0.521 | 78 |
| 28 | 0118OS | 300 | 2.5 | 0.500 | 81 |
| 29 | 0119OD | 300 | 2.5 | 0.500 | 54 |
| 30 | 0119OS | 300 | 2.5 | 0.500 | 54 |

**Supplementary Table 3.** Opening mode test without notch specimen details

| **S. No.** | **Specimen** | **Strain Rate** | **Thickness** | **Age** |
| --- | --- | --- | --- | --- |
|  |  | **(mm/min)** | **(mm)** | **(Years)** |
| 1 | 1850OS | 3 | 0.500 | 81 |
| 2 | 2018OD | 3 | 0.518 | 51 |
| 3 | 2022OS | 3 | 0.524 | 89 |
| 4 | 2130OD | 3 | 0.536 | 35 |
| 5 | 2185OS | 30 | 0.527 | 21 |
| 6 | 1851OS | 30 | 0.500 | 83 |
| 7 | 1884OS | 30 | 0.526 | 52 |
| 8 | 1992OD | 30 | 0.516 | 54 |
| 9 | 1997OD | 30 | 0.534 | 25 |
| 10 | 2980OD | 30 | 0.528 | 65 |
| 11 | 1868OS | 300 | 0.526 | 61 |
| 12 | 1884OD | 300 | 0.526 | 52 |
| 13 | 1925OD | 300 | 0.534 | 62 |
| 14 | 1952OD | 300 | 0.534 | 56 |
| 15 | 1997OS | 300 | 0.500 | 25 |


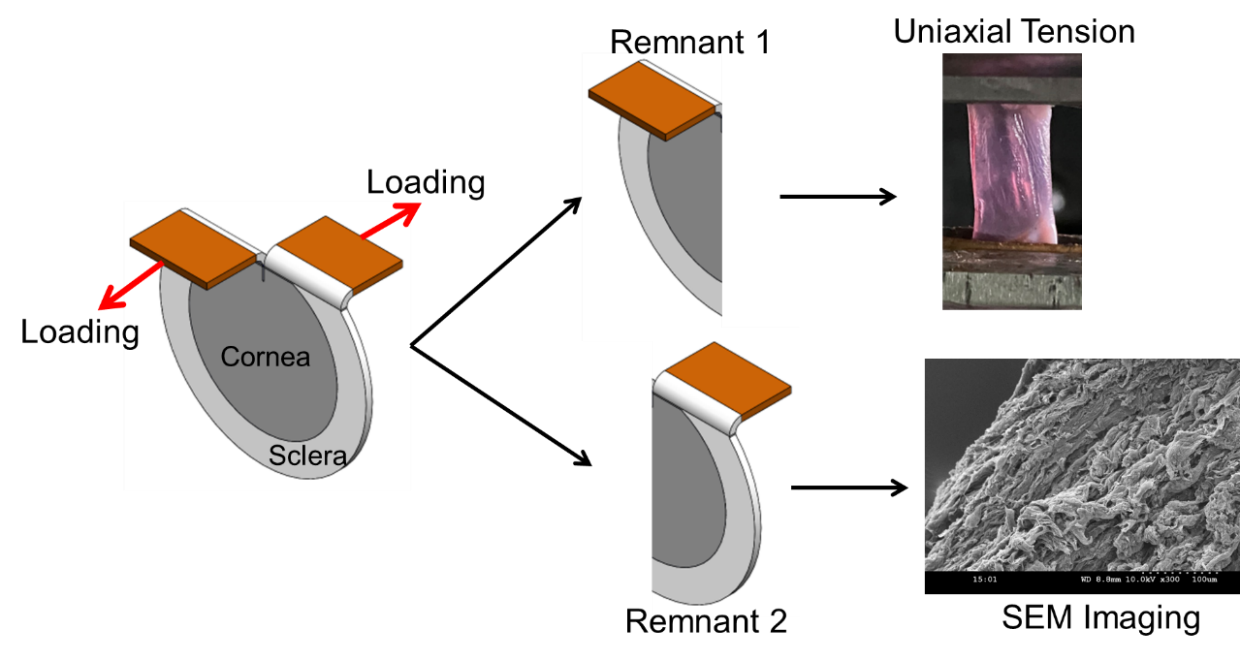


**Supplementary Figure 1.** Trouser tear test specimens resulting in 2 remnants and their further use for uniaxial tension and SEM imaging.

**Methodology of curve fitting for tensile test data:**

The tangential stiffness of the cornea is obtained at 0.3% strain ($\epsilon$) by fitting the EXP2P function in OriginPro 9.0 relating stress ($\sigma$) and strain ($\epsilon$) given by $\sigma=a.b^{\epsilon}$. The tangential stiffness at a known strain ($\epsilon_{o}$= 3% or 0.03) is given by the following expression,

$$E_{t}:\left( \epsilon_{o}:0.03 \right)=\frac{d\sigma}{d\epsilon}=a.\left( \ln b \right).b^{\epsilon_{o}}$$

**Supplementary Table 4.** Stress strain data for specimens of 3 mm strain rate.

| **Specimen** | **Coefficient, a** | **Coefficient, b** | **ɛ** | **b(ɛ)** | **a.b(ɛ)** | **lnb** | **Tangential stiffness** | **Toughness** |
| --- | --- | --- | --- | --- | --- | --- | --- | --- |
| **0255OD** | 0.06499 | 30128900 | 0.03 | 1.676368526 | 0.10894719 | 17.2209954 | 1.876 | 7.16315 |
| **0274OD** | 0.01497 | 873800.1642 | 0.03 | 1.507448082 | 0.022566498 | 13.68060698 | 0.309 | 7.308 |
| **0277OD** | 0.00395 | 1.26936E+11 | 0.03 | 2.153314894 | 0.008505594 | 25.56694886 | 0.217 | 5.324 |
| **0292OD** | 0.06045 | 54273700 | 0.03 | 1.706230429 | 0.103141629 | 17.80955032 | 1.837 | 5.324 |
| **0299OD** | -0.40103 | 5.04966E-54 | 0.03 | 0.025182443 | -0.010098915 | -122.7202741 | 1.239 | 4.69498 |
| **0299OS** | 0.02587 | 15393000 | 0.03 | 1.642932414 | 0.042502662 | 16.54942342 | 0.703 | 5.43191 |
| **0311OS** | 0.04498 | 6123580000 | 0.03 | 1.966120638 | 0.088436106 | 22.53541273 | 1.993 | 7.556 |
| **0345OD** | 0.00901 | 10793900000 | 0.03 | 1.999840465 | 0.018018563 | 23.102247 | 0.416 | 5.12138 |
| **0385OD** | 0.0518 | 630562.3516 | 0.03 | 1.492766365 | 0.077325298 | 13.35436732 | 1.033 | 4.27536 |
| **0426OD** | 0.06131 | 5910870 | 0.03 | 1.596428757 | 0.097877047 | 15.59230359 | 1.526 | 7.068 |

**Supplementary Table 5.** Stress strain data for specimens of 30 mm strain rate.

| **Specimen** | **Coefficient, a** | **Coefficient, b** | **ɛ** | **b(ɛ)** | **a.b(ɛ)** | **lnb** | **Tangential stiffness** | **Toughness** |
| --- | --- | --- | --- | --- | --- | --- | --- | --- |
| **0395OS** | 0.00528 | 58352900000 | 0.03 | 2.103690634 | 0.011107487 | 24.78977489 | 0.275352091 | 6.955 |
| **0497OS** | 0.00872 | 27546600000 | 0.03 | 2.056847249 | 0.017935708 | 24.03914495 | 0.431159085 | 6.152 |
| **0578OD** | 0.00803 | 1768970000 | 0.03 | 1.894225218 | 0.015210628 | 21.29366329 | 0.323890002 | 6.517 |
| **0586OD** | 0.18006 | 799357.7492 | 0.03 | 1.50342662 | 0.270706997 | 13.59156387 | 3.679331443 | 6.118 |
| **0589OD** | 0.04225 | 317213.1246 | 0.03 | 1.462313652 | 0.061782752 | 12.66732914 | 0.782622452 | 7.196 |
| **0590OD** | 0.00791 | 6.86613E+12 | 0.03 | 2.427176511 | 0.019198966 | 29.55762174 | 0.567475781 | 6.195 |
| **0594OD** | 0.0131 | 7216710000 | 0.03 | 1.97583274 | 0.025883409 | 22.69966501 | 0.587544711 | 7.401 |
| **0594OS** | 0.01299 | 1385480000 | 0.03 | 1.880390324 | 0.02442627 | 21.04931249 | 0.514156197 | 6.692 |
| **0610OD** | 0.000195013 | 1.65978E+26 | 0.03 | 6.117887911 | 0.001193068 | 60.37389748 | 0.072030146 | 6.652 |
| **3007OD** | 0.2098 | 1.69E+06 | 0.03 | 1.537494081 | 0.322566258 | 14.33846236 | 4.625104152 | 6.791 |

**Supplementary Table 6.** Stress strain data for specimens of 30 mm strain rate.

| **Specimen** | **Coefficient, a** | **Coefficient, b** | **ɛ** | **b(ɛ)** | **a.b(ɛ)** | **lnb** | **Tangential stiffness** | **Toughness** |
| --- | --- | --- | --- | --- | --- | --- | --- | --- |
| **0655OS** | 0.10498 | 3072290 | 0.03 | 1.565394726 | 0.164335138 | 14.93793377 | 2.454827412 | 7.45909 |
| **0615OD** | 0.01979 | 7.3498E+11 | 0.03 | 2.269803522 | 0.044919412 | 27.32310912 | 1.227337988 | 5.64573 |
| **0586OS** | 0.19038 | 875794.0949 | 0.03 | 1.507551164 | 0.287007591 | 13.68288629 | 3.927092227 | 5.39217 |
| **0583OD** | 0.01628 | 916644000 | 0.03 | 1.857231414 | 0.030235727 | 20.63622973 | 0.623951417 | 7.60127 |
| **0578OS** | 0.06849 | 3732020 | 0.03 | 1.574556753 | 0.107841392 | 15.1324602 | 1.631905573 | 6.06137 |
| **0563OD** | 0.16409 | 6344290 | 0.03 | 1.599821359 | 0.262514687 | 15.66306575 | 4.111784802 | 9.05152 |
| **0391OS** | 0.03665 | 529461000 | 0.03 | 1.826901024 | 0.066955923 | 20.08737007 | 1.344968394 | 8.08361 |
| **0273OD** | 0.0299 | 976572000 | 0.03 | 1.860763284 | 0.055636822 | 20.69955904 | 1.151657685 | 6.83429 |
| **0655OD** | 0.07926 | 9079510 | 0.03 | 1.6171186 | 0.12817282 | 16.02153078 | 2.053524785 | 7.09597 |
| **1800OS** | 0.009156 | 12640000000 | 0.03 | 2.009335292 | 0.018397474 | 23.26013223 | 0.427927676 | 6.989 |
